# RB/PLK1-dependent induced pathway by SLAMF3 expression inhibits mitosis and control hepatocarcinoma cell proliferation

**DOI:** 10.18632/oncotarget.6954

**Published:** 2016-01-20

**Authors:** Hicham Bouhlal, Hakim Ouled-Haddou, Véronique Debuysscher, Amrathlal Rabbind Singh, Christèle Ossart, Aline Reignier, Hakim Hocini, Gregory Fouquet, Mohammed Al Baghami, Mélanie Simoes Eugenio, Eric Nguyen-Khac, Jean-Marc Regimbeau, Ingrid Marcq

**Affiliations:** ^1^ Centre Universitaire de Recherche en Santé CURS, CAP-Santé (FED 4231), Université de Picardie Jules Verne, CHU Sud, Amiens, France; ^2^ Service d'Hématologie Clinique et de Thérapie Cellulaire Centre Hospitalier Universitaire Sud, Amiens, France; ^3^ IMRB, Equipe 16, Génomique Médicale, UFR de Médecine, Créteil, France; ^4^ Service Hepato-Gastroenterologie, Centre Hospitalier Universitaire Sud, Amiens, France; ^5^ Service de Chirurgie Digestive Centre Hospitalier Universitaire Sud, Amiens, France

**Keywords:** SLAMF3, HCC, retinoblastoma factor RB, polo-like kinase 1 (PLK1), mitosis

## Abstract

Polo-like kinase PLK1 is a cell cycle protein that plays multiple roles in promoting cell cycle progression. Among the many roles, the most prominent role of PLK1 is to regulate the mitotic spindle formation checkpoint at the M-phase. Recently we reported the expression of SLAMF3 in Hepatocytes and show that it is down regulated in tumor cells of hepatocellular carcinoma (HCC). We also show that the forced high expression level of SLAMF3 in HCC cells controls proliferation by inhibiting the MAPK ERK/JNK and the mTOR pathways. In the present study, we provide evidence that the inhibitory effect of SLAMF3 on HCC proliferation occurs through Retinoblastoma (RB) factor and PLK1-dependent pathway. In addition to the inhibition of MAPK ERK/JNK and the mTOR pathways, expression of SLAMF3 in HCC retains RB factor in its hypophosphorylated active form, which in turn inactivates E2F transcription factor, thereby repressing the expression and activation of PLK1. A clear inverse correlation was also observed between SLAMF3 and PLK expression in patients with HCC. In conclusion, the results presented here suggest that the tumor suppressor potential of SLAMF3 occurs through activation of RB that represses PLK1. We propose that the induction of a high expression level of SLAMF3 in cancerous cells could control cellular mitosis and block tumor progression.

## INTRODUCTION

Hepatocellular carcinoma (HCC) is a highly aggressive cancer, which results in more than 600,000 deaths every year worldwide [1]. Although the major risk factors of HCC have been identified which includes the infection with hepatitis B or C viruses [2], the balance between cell cycle regulators and cell proliferation is also an important determinant of tumor development and/or behavior [3]. Although the expression of pro-apoptotic genes is decreased in HCC, the balance between death and survival in HCC is dysregulated due to over-activation of anti-apoptotic pathways. Indeed, some molecules involved in counter-acting apoptosis, such as Bcl-XL, Mcl-1, c-IAP1, XIAP and survivin are known to be overexpressed in HCC cells. Furthermore, some growth factors that mediate cell survival are also up-regulated in HCC, as well as the molecules involved in the cleavage of their proforms to an active form. The expression and/or activation of the JAK/STAT, PI3K/AKT and RAS/ERKs pathways are also reported to be enhanced in HCC cells, conferring on them resistance to apoptotic stimuli [4–8].

SLAMF3 belongs to signaling lymphocytic activation molecule family of receptors (SLAMF-Rs) that trigger both inhibitory and activation signals in immune cells [9]. Recently, we identified the expression of SLAMF3 in hepatocytes [10]. We demonstrated a link between high level expression of SLAMF3 in HCC cells and low proliferation index. SLAMF3 overexpression inhibited ERK1/2, JNK and mTOR pathways and reduced tumor progression of HCC xenografts in a mouse model [10]. The identification of this new molecule in hepatocytes and its role in controlling the proliferation of HCC cells prompted us to investigate other potential pathways involved in the anti-proliferative effect of SLAMF3. In a preliminary study, we analyzed the mRNA and quantified the expression of genes implicated in proliferation and cell cycle control in the SLAMF3 overexpressing cells. Among the analyzed genes, the transcripts of Polo-like kinase PLK1 were found to be significantly decreased. The levels of PLK1 mRNA, coding for a serine-threonine kinase, vary dramatically during cell cycle progression. The levels range from very low or undetectable amounts in G0–G1, steadily increasing amounts have been detected during S phase onwards, and to a peak in G2-M phase [11, 12]. The most prominent role of PLK1 is to regulate the spindle checkpoint in the M-phase [13]. It has also been observed that mutation in Plk1 alleles disrupts the spindle formation resulting in polyploid cells [14]. Comparison of PLK1 expression in HCC tumors and their corresponding tumor free tissue showed that it is overexpressed in the tumoral tissue. High-level expression of PLK1 in HCC tumoral tissue correlated with low overall survival rate. In addition, siRNA against PLK1 increased apoptosis in Huh-7 cell line in a caspase-independent manner and induced tumor regression in siPLK1-treated mice [15]. PLK1 depletion leading to G2/M arrest, inhibition of cell proliferation and promotion of apoptosis via downregulation of Survivin expression has also been reported [16]. RB activity is responsible for repression of the PLK1 promoter, which in turn depends on the activity of SWI/SNF chromatin remodeling complex [17]. The extended RB pathway comprises of p16^INK4a^ and p21^CIP1^ family members, which inhibit the kinase activity of Cyclin–Cyclin-dependent kinase (CDK) complexes; these complexes in turn inactivate the RB protein and its two other family members p107 and p130 by hyperphosphorylation during G1/S transition of the cell cycle, thereby activating E2F transcription factors [18, 19]. Multiple events resulting in the functional inactivation of RB pathway in human HCC occur early in the course of the disease, suggesting that the RB pathway plays a pivotal role in preventing initiation of HCC [20, 21].

In the present study, we report the regulatory effect of hepatocyte SLAMF3 on PLK1 expression via the RB pathway. We provide evidence that the anti-proliferative effect of hepatocyte SLAMF3 is, in part, through the inhibition of PLK1 expression and activity. We also report an inverse correlation between the high level expression of PLK1 and low expression levels of SLAMF3 in HCC patients suggesting an anti-mitotic role of SLAMF3 through a PLK1-dependent pathway. Taken together, the induction of high level expression of hepatocyte SLAMF3, an anti-mitotic factor could be a potent strategy for the therapeutic intervention in HCC.

## RESULTS

### Overexpression of SLAMF3 blocks HCC cells proliferation

**Figure 1:**
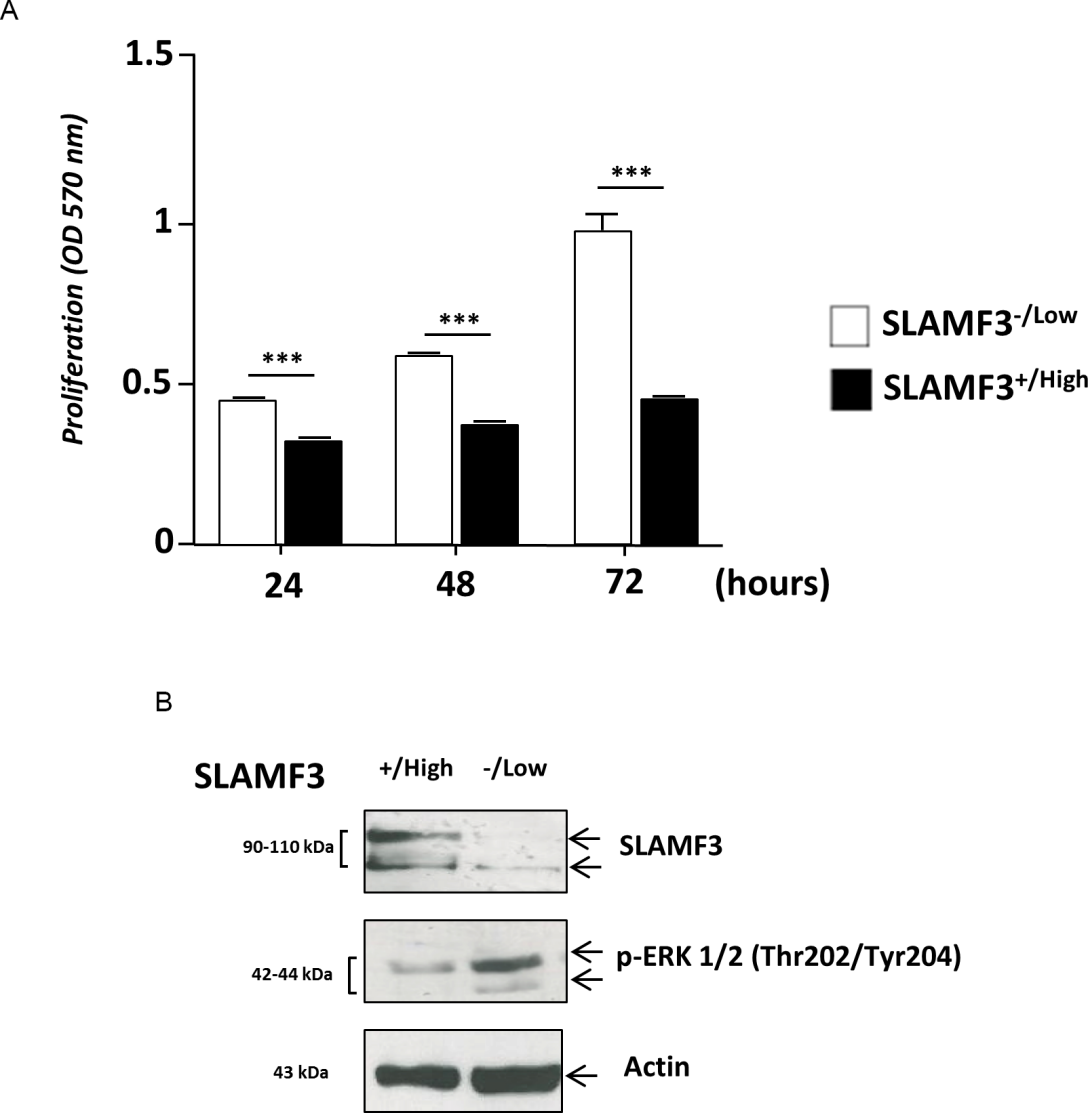
Hepatocyte SLAMF3 forced expression decreased HCC cells proliferation (**A**) Proliferation was determined by MTT test after 24, 48 and 72 h cultures of sorted SLAMF3^+/high^ and SLAMF3^−/low^ sub-populations after the introduction of SLAMF3 coding plasmid in Huh-7 cell line. The results are presented as the mean SD of three independent experiments (*n* = 3; ***p < 0.005). (**B**) SLAMF3 expression level and phospho-ERK1/2 proteins were detected by Western blot analysis in SLAMF3^+/high^ and SLAMF3^−/low^ sub-populations of Huh-7 cells with actin used as control. One representative result from 3 independent experiments is shown here.

We have recently identified and described the expression of SLAMF3 receptor in hepatocytes [10] and shown that the high level expression of SLAMF3 inhibits proliferation in HCC cells. The cancerous wild type Huh-7 and HepG2 cell lines do not express more than 5–10% of SLAMF3 in the cell surface [10] in comparison to primary hepatocytes. Transient transfection of cell lines with plasmid increased the expression of SLAMF3 and yielded 60–70% positive cells. SLAMF3^+/high^ and SLAMF3^−/low^ cells were sorted and observed that the enhanced SLAMF3 expression reduced cell proliferation by 50% at 72 h post-transfection (Figure 1A). As described previously, the high level expression SLAMF3 reduced the phosphorylation of MAPK ERK 1/2 as shown in Figure 1B. In a similar manner SLAMF3 was also overexpressed in another HCC cell line HepG2 and observed the inhibition of ERK1/2 as observed in Huh-7. However the cell proliferation even though inhibited upon overexpression of SLAMF3 was only 40% as compared to 50% inhibition observed in Huh-7. Inhibition of ERK1/2 and reduction in cell proliferation observed both in Huh-7 and HepG2 confirmed the anti-proliferative effect of SLAMF3 in HCC cells (Supplementary Figure 1A, 1B).

### SLAMF3 overexpression in HCC leads to increased cell size and granularity

**Figure 2:**
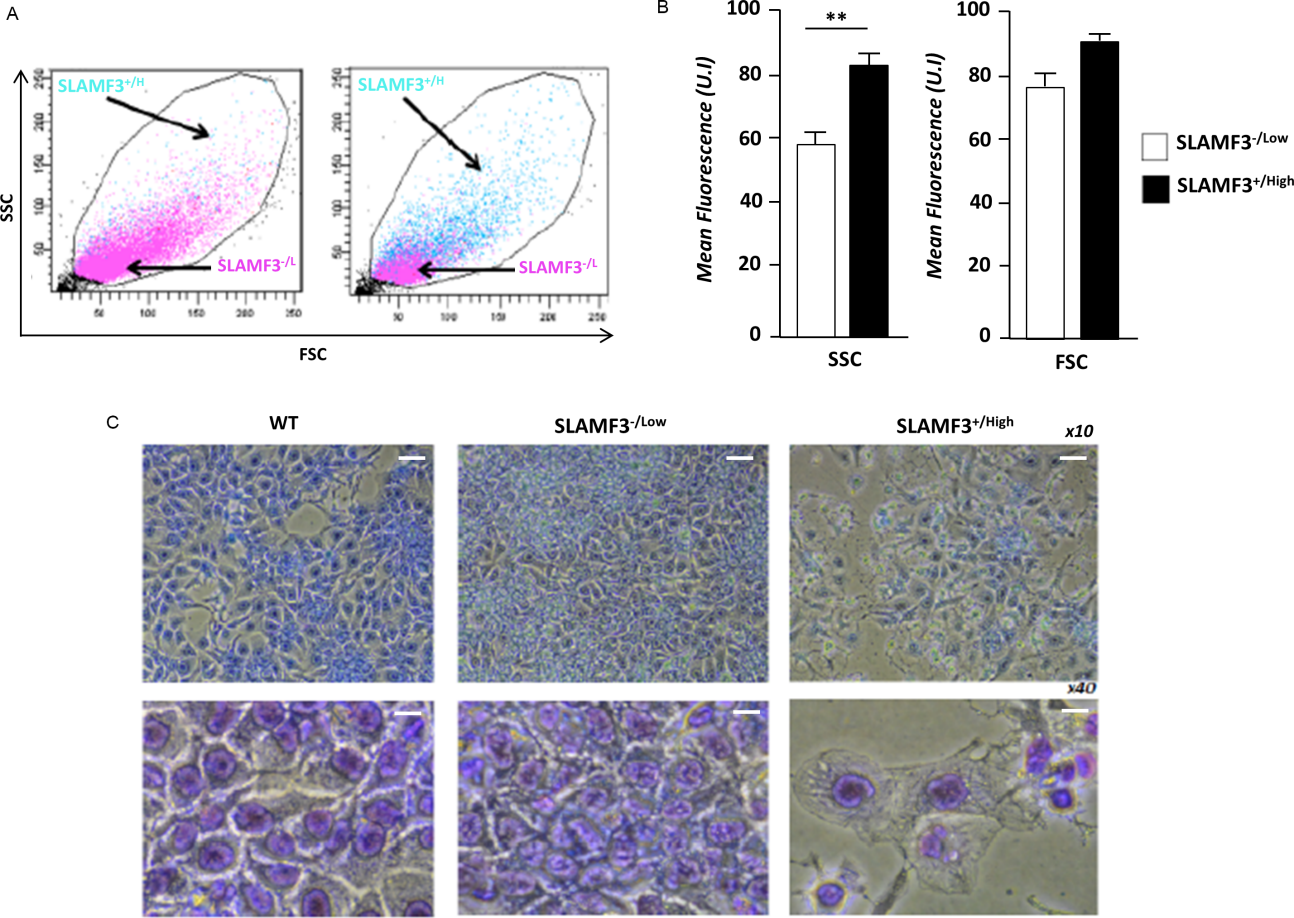
SLAMF3 overexpression induced morphologic changes in HCC cells (**A**) Forward and side scatter (FCS-SSC) cytometery analysis of SLAMF3^+/high^ and SLAMF3^−/low^ gated cells (green and rose, respectively). (**B**) The mean fluorescence of FSC and SSC in SLAMF3^−/low^ and SLAMF3^+/high^ sub-populations were determined from three independent experiments (*n* = 3; **p < 0.01). (**C**) Huh-7 cells were transfected with SLAMF3 and sorted as SLAMF3^+/high^ and SLAMF3^−/low^ and cell morphology, by Giemsa staining, was compared to that of WT cells cultures. Morphologic analysis was determined at 48 hours after SLAMF3 transfection. One representative from two independent experiments is presented as microscopy analysis at 10x and 40x.

Huh-7 cells overexpressing SLAMF3 were compared to cells transfected with control plasmid (mock) and tested for their size and granularity by forward and side scatter in a flow cytometer. We show that SLAMF3 overexpressing cells present a bigger cell size and intense granulation (Figure 2A). Indeed, the cell sizes were significantly increased by 30% in SLAMF3^+/high^ compared to SLAMF3^−/low^ (Figure 2B). MGG staining showed that Huh-7 cells overexpressing SLAMF3 have an enhanced cytoplasm with denser chromatin when compared to SLAMF3^−/low^ (Figure 2C).

### SLAMF3 expression induces cell cycle arrest at G2/M

**Figure 3:**
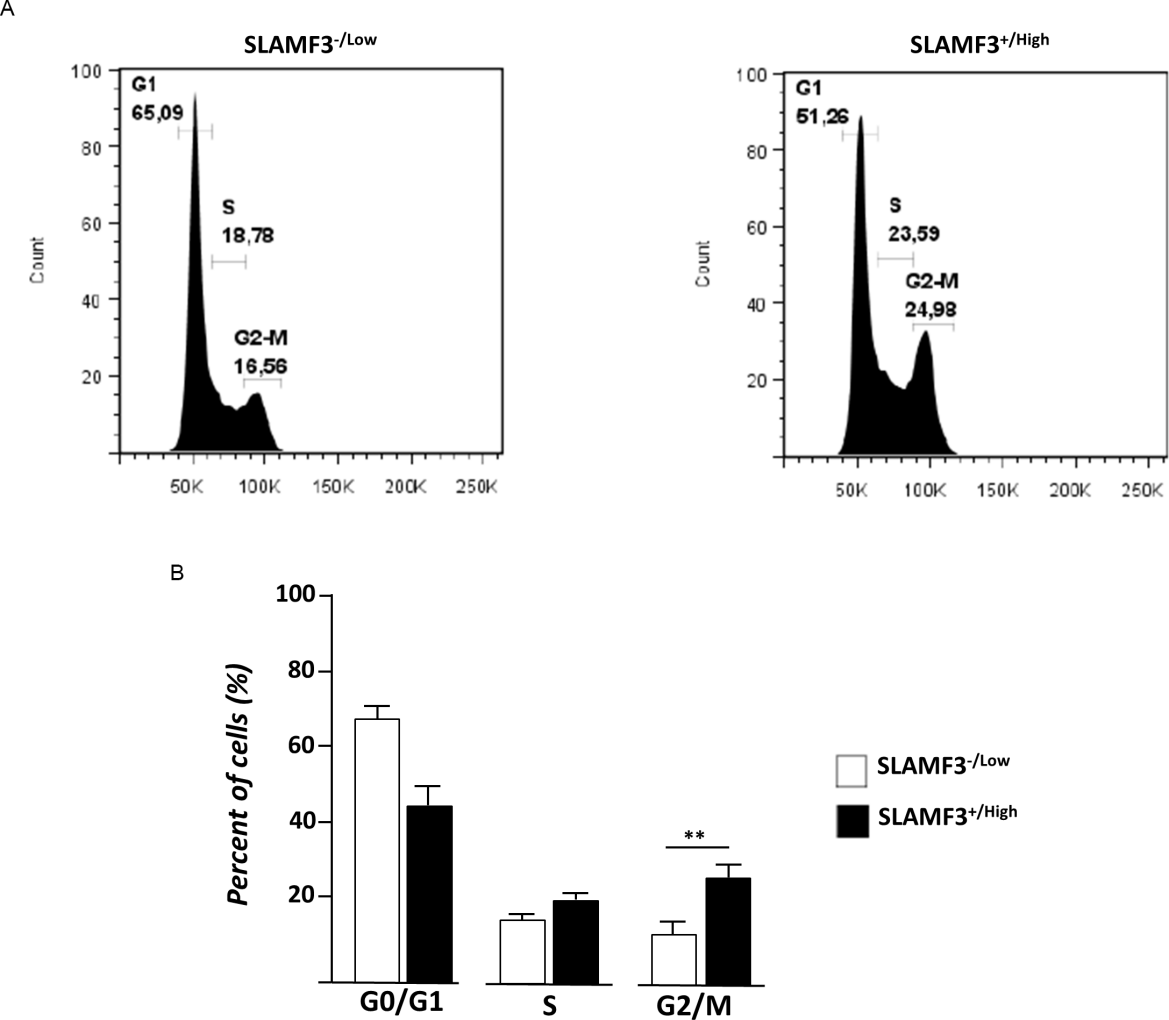
SLAMF3 expression induced cell cycle arrest and activation of the G2 checkpoint (**A**) Cell-cycle was analyzed at 48 h after SLAMF3 induced expression in cultured SLAMF3^+/high^ and SLAMF3^−/low^ cells. Percent of cells (%) at G1, S and G2/M stages of cell cycle is indicated on graphics from one representative experiment from three independent (*n* = 3). (**B**) The mean of cell distribution in each cell cycle stages was presented as mean +/− SD (*n* = 3; *p < 0.01*).

We have previously shown that overexpression of SLAMF3 in cancerous cells leads to the inhibition of MAPK ERK/JNK, mTOR phosphorylation and induces apoptosis by a caspase-dependent-pathway [10]. These observations prompted us to analyze the effect of the signal induced by the high expression level of SLAMF3 on the cell cycle. Huh-7 cells were transfected with SLAMF3 plasmid and sorted for SLAMF3^+/high^ and SLAMF3^−/low^ sub-populations 48 hours after transfection to test the cell cycle distribution in SLAMF3^+/high^ and compare to SLAMF3^−/low^ sub-population. In SLAMF3^+/high^ subpopulation net cell cycle arrest was observed with accumulation of cells at G2/M stage (*p < 0.01*) and less pronounced at DNA synthesis phase (S-phase). SLAMF3^−/low^ cells remain predominantly in G0/G1 (Figure 3A, 3B).

### Overexpression of SLAMF3 inhibits expression and phosphorylation of PLK1

**Figure 4:**
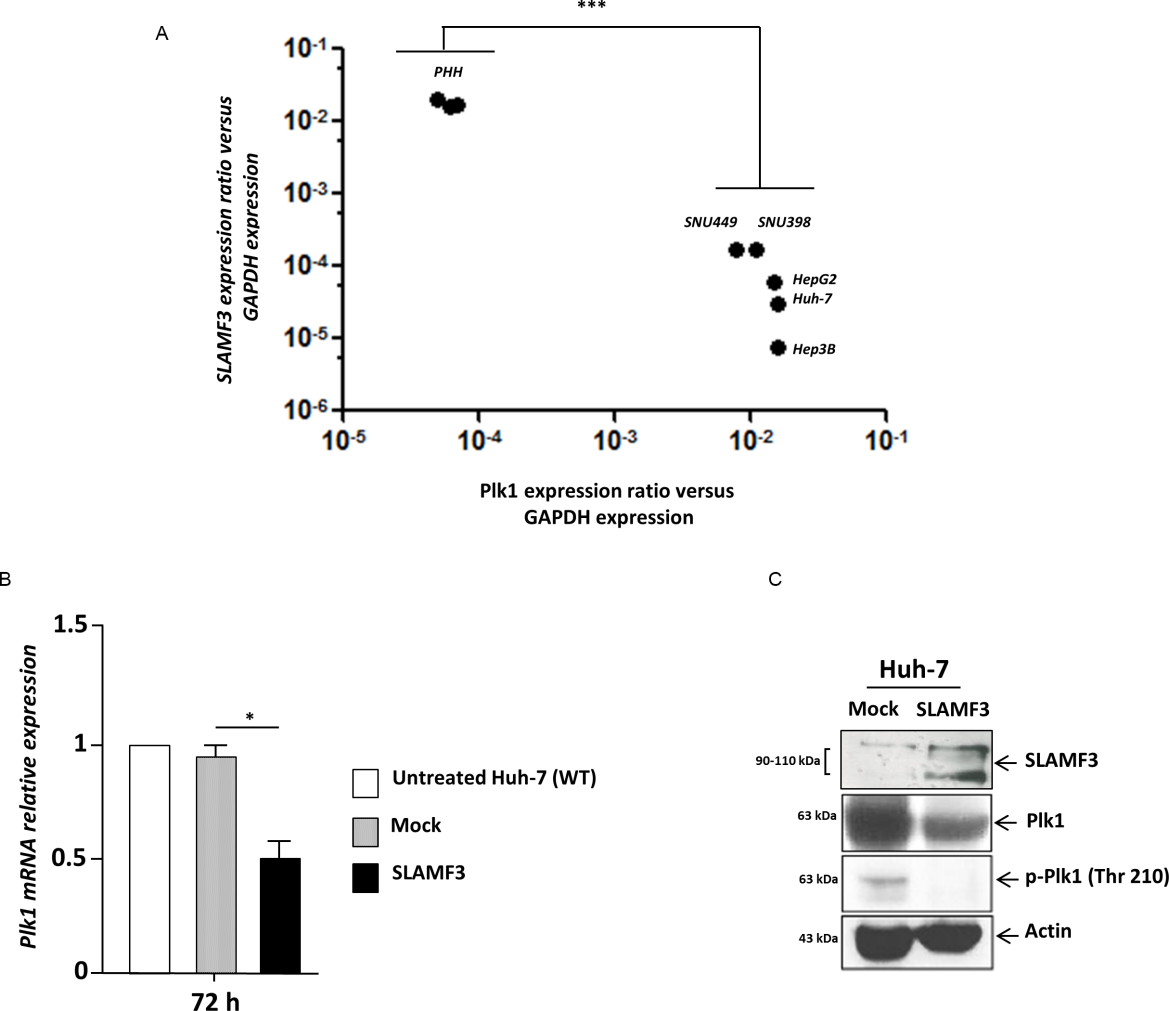
Restoration of SLAMF3 high expression in HCC cells inhibits PLK1 expression and activation (**A**) SLAMF3 and PLK1 mRNA was quantified in healthy primary human hepatocytes (PHH) and in HCC cell lines (Huh-7, HepG2, Hep3B, SNU 398 and SNU 449). Transcripts numbers were standardized by GAPDH quantification used as control. Results are presented as the mean of six independent experiments +/− SD (*n* = 6, ***p < 0.001). (**B**) PLK1 mRNA expression quantified in SLAMF3 overexpressing Huh-7 cells (SLAMF3) compared to Mock (free-plasmid treated cells) and with untreated cells. Results presented as the mean of six independent experiments (*n* = 6, *p < 0.05). (**C**) PLK1 and phospho-Plk-1 (p-PLK1, Thr 210) proteins were detected by WB analysis in Huh-7 cells overexpressing SLAMF3 and in mock cells at 48 hours. Detection of actin was used as control. One representative from three independent experiments is shown here.

Subsequently, based on increased cytoplasmic content, nuclei size and observed cell cycle blockade in presence of high SLAMF3 expression, we hypothesized that the expression of SLAMF3 blocks cell division after DNA replication. Polo-like kinase-1 PLK-1 is one of the crucial factors involved in the regulation of mitosis. This protein is involved in the mitotic spindle formation and separation of two daughter cells at late mitosis stage. First, we compared PLK-1 and SLAMF3 expression in HCC and healthy primary hepatocytes. We highlight significant inverse correlation (*r =* −*0.9701, p < 0.0005*) between PLK-1 and SLAMF3 expression (Figure 4A). All HCC cells lines expressed very low levels of SLAMF3 compared to healthy hepatocytes PHH. Among the HCC cells, Huh-7, HepG2 and Hep3B cell lines expressed lower levels of PLK1 than SNU398 and SNU449.

Huh-7 cells were transfected transiently to overexpress SLAMF3 and the PLK1 mRNA was quantified by qPCR. It was observed that overexpression of SLAMF3 significantly (*p* < 0.05) inhibited the expression of PLK1 mRNA (Figure 4B). Western blot analysis also showed that SLAMF3 overexpression reduced the expression of PLK1. Western blot performed using anti-phospho PLK1 antibody showed that the overexpression of SLAMF3 also reduced the activation of PLK1 (Figure 4C).

### Hepatocyte SLAMF3 maintains RB in its activated form and suppresses PLK1- dependent mitosis

**Figure 5:**
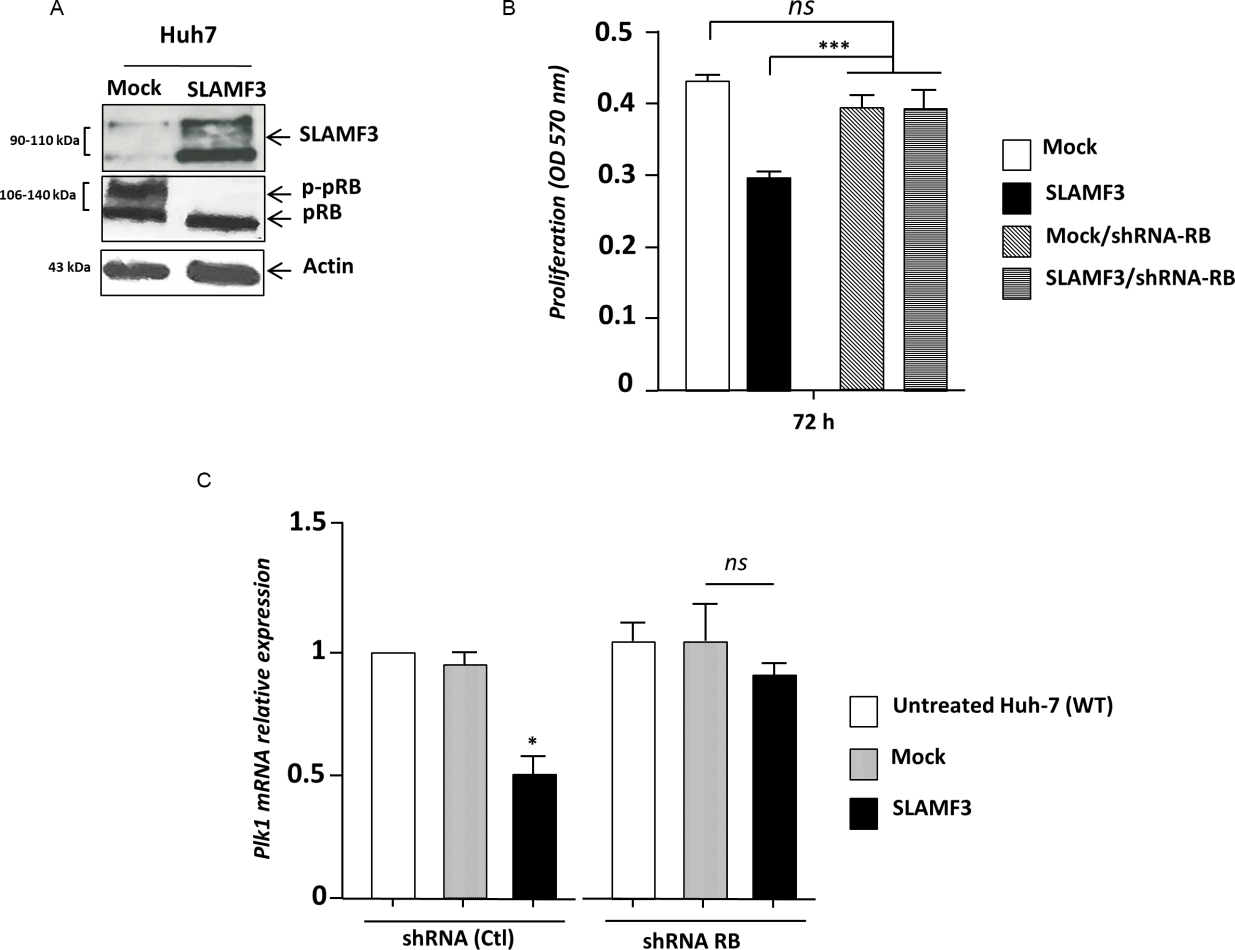
SLAMF3 high expression activates RB (**A**) Hypo and hyper-phosphorylated forms of RB (pRB and p-pRB, respectively) were detected by WB analysis in Huh-7 cells overexpressing SLAMF3 and in mock cells at 48 hours post-transfection. (**B**) Proliferation of cells was determined by MTT test after 24, 48 and 72 h of culture of cells from Huh-7 WT and Huh-7 stably transfected by shRNA specifically directed against RB in both conditions when cells overexpressed SLAMF3 and mock (Mock *vs* SLAMF3). The results were presented as the mean +/− SD (*n* = 3; ***p < 0.005, ns: statistically non significant). (**C**) PLK1 transcripts were quantified in shRNA RB and shRNA control-treated Huh-7. In both conditions, cells were untransfected (untreated cells), transfected with free-plasmid (Mock) and with SLAFM3 coding plasmid (SLAMF3). Results presented as the mean from six independent experiments (*n* = 6, *p < 0.05, *ns: statistically non significant)*.

The Retinoblastoma RB factor is one of the many factors, which control the expression of PLK1 [17]. The hyperphosphorylation of RB results in its detachment from E2F-suppressor complex that induces the expression of genes under control of RB/E2F complex. Inversely, the hypophosphorylated form of RB remains attached to the E2F factor and represses the expression of genes under the control of RB [10, 17]. The overexpression of SLAMF3 in Huh-7 cells drastically decreased the hyper-phosphorylated form (p-pRB) where as both hypo and hyper-phosphorylated forms were present in the mock (Figure 5A). This result suggests that overexpression of SLAMF3 retains RB in its active form that remains potentially fixed to the E2F-suppressor complex. To verify the link between SLAMF3 and RB, RB specific shRNA was introduced in Huh-7 cells to create a stably transfected cell line. Expression levels of RB was tested in the cell line and observed that the introduction of RB specific shRNA leads to 70% reduction in the mRNA and 80% reduction in the protein (see Supplementary Figure 2A, 2B). To understand the role of RB in the anti-proliferative property of SLAMF3, Huh-7/shRNA-RB cells were transiently transfected to over express SLAMF3 and the proliferation was tested by MTT assay. The results show that the overexpression of SLAMF3 did not have any effect on the cell proliferation suggesting that the inhibitory effect of SLAMF3 is mediated by RB factor (Figure 5B). In addition, the inhibitory effect of SLAMF3 on PLK-1 expression was decreased in absence of RB (Figure 5C), suggesting a strong link between the SLAMF3 overexpression, activation of RB, that by its hypophosphorylation in turn negatively regulates the expression and activation of PLK1 resulting in cell cycle arrest at mitosis stage.

### Expression level of SLAMF3 inversely correlates with PLK1 expression in patients with HCC

**Figure 6:**
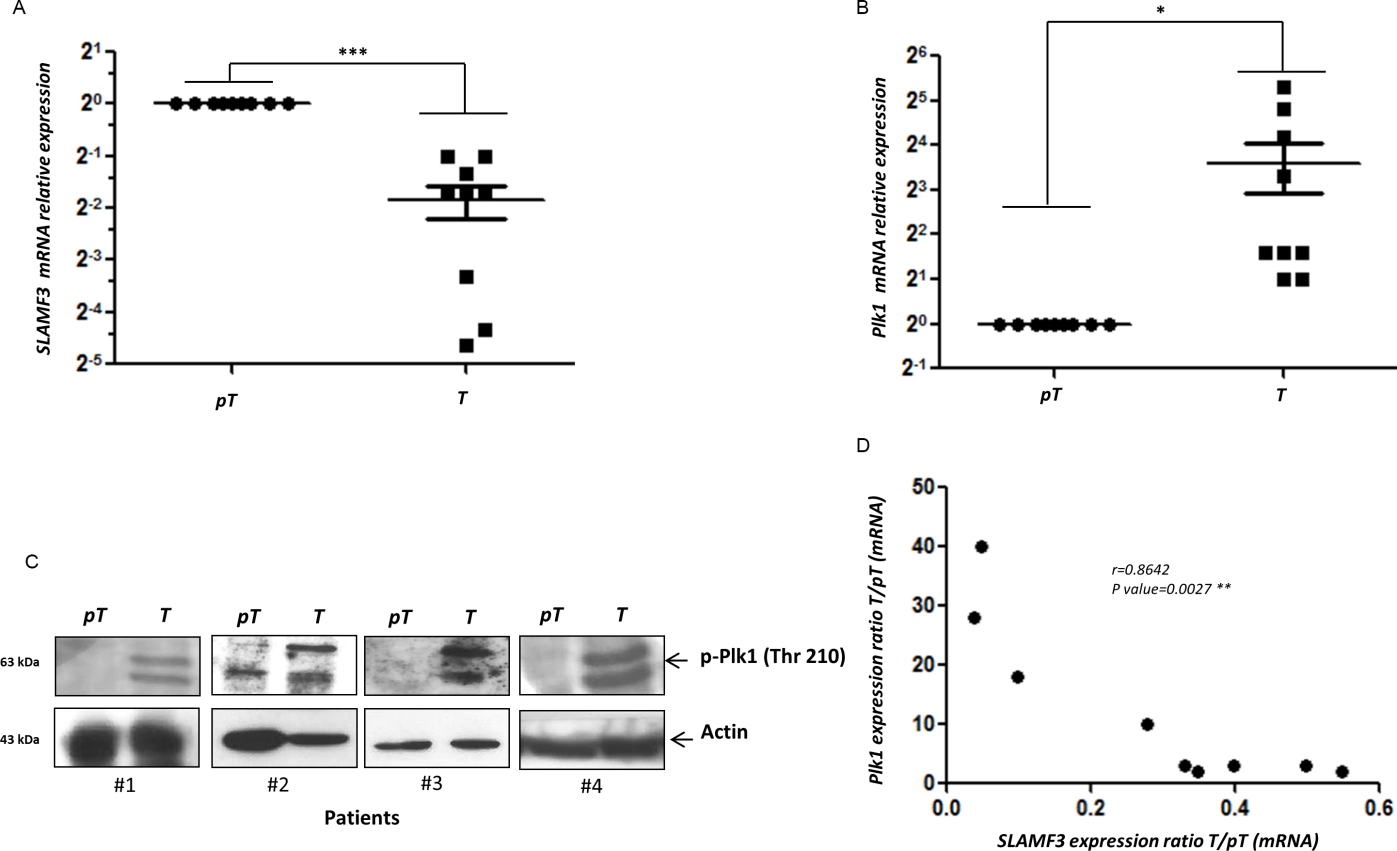
PLK1 and SLAMF3 expression level in HCC patients (**A**) Quantification of SLAMF3 mRNA expression in HCC patient samples. (**B**) Quantification of PLK1 mRNA expression in HCC patient samples. (**C**) Levels of PLK1 protein in tumour and peri-tumour samples of HCC patients detected by western blot. (**D**) Inverse correlation between SLAMF3 and PLK1 mRNA.

To compare the expression levels of PLK1 mRNA between HCC and healthy adjacent normal tissue from the same patient, total RNA was extracted and real time RT-PCR was performed on the samples. Thirteen pairs (*n* = 13) of paired resections samples (T/pT) obtained from surgery department (CHU, Amiens, France). Analysis of SLAMF3 mRNA in samples demonstrated that in nine samples (9/13, 70%), SLAMF3 mRNA in HCC (T) tissues was significantly lower than those of adjacent normal tissue (pT) (Figure 6A and Supplementary Figure 3A; *p < 0.005*). Four samples (4/13; 30%) presented higher SLAMF3 mRNA expression in T than in pT. This paradoxical result compared to our previous observations prompted us to check the presence of other cell types that express the SLAMF3 mRNA there by increasing the expression of this molecule in hepatic tumor tissue. Indeed, we quantified transcripts of CD3 and CD64, specific marker for T lymphocytes and macrophages, respectively, which are described as SLAMF3 expressing cells [22]. We show that the four samples which presented high SLAMF3 in tumor samples, also expressed, high levels of CD3 and CD64 suggesting infiltration of immune cells in tumor tissue whereas no CD3 and CD64 transcripts were detected in corresponding pT samples (Supplementary Figure 4A, 4B).

The mRNA quantification also showed that HCC tissues expressed, significantly (*p* < 0.05), high levels of PLK1 mRNA than adjacent normal tissue (Figure 6B and Supplementary Figure 3B). These results were also confirmed by western blot analysis in T and pT samples from patient #1, #2, #3 and #4 (Figure 6C). We observed a significant inverse correlation between SLAMF3 and PLK1 expression in the patients (*p < 0.005; r = 0.86*) (Figure 6D).

## DISCUSSION

PLK1 has been shown to be intimately involved in spindle formation and chromosome segregation during mitosis and therefore plays an important role in the regulation of cell cycle [23–26]. In HCC, the overexpression of PLK1 correlates with low overall survival rate in HCC patients. The *in vitro* introduction of siRNA against PLK1 in Huh-7 cells increased apoptosis in caspase-independent pathway and induced tumor regression in siPLK1-treated mice [15]. The levels of PLK1 increased during S phase and reached peak at mitosis G2-M and its activity is elevated in tissues and cells having a high mitotic index, that includes cancerous cells [12]. The depletion of PLK1 led to G2/M arrest, inhibition of cell proliferation and apoptosis promotion via down-regulation of survivin expression [16]. Based on these findings, PLK1 has been proposed as a novel diagnostic marker for cancer, and its inhibition might represent a rewarding approach in cancer therapy [27]. Indeed, several PLK1 inhibitors, including BI2536 and GSK461364, are in clinical studies for treating patients with various cancers [28]. Volasertib (BI6727) was the first PLK1 inhibitor to be tested clinically has reached phase III for the treatment of acute myeloid leukemia (AML). In addition to its proven activity, when used alone, Volasertib increased anti-leukemic activity in AML when combined with low-dose cytarabine compared with cytarabine alone [29]. In the present work, we highlight the link between SLAMF3 expression in HCC cells and the increased size of nucleus as well as the enhancement of cytoplasm. We previously reported that the increase in the cell size in SLAMF3 overexpressing cells continues with induction of apoptosis by a caspase-dependent pathway [10]. Transcriptomic screening of SLAMF3 overexpressing cells identified that PLK1, a major target of RB was repressed. Overexpression of SLAMF3 inhibits the expression of PLK1 by more than 50%. The inhibition of PLK1 transcripts is reinforced by total inhibition of PLK1 phosphorylation when SLAMF3 is overexpressed. Taken together, our results provide evidence that SLAMF3 abolishes the expression and activation of PLK1. In addition we also show that SLAMF3 overexpression induces cel1 cycle arrest at S-G2/M. The transition at the G2/M stage of cell cycle depends on the action of cyclins A/B and CDK1. The CDK1 activation in turn depends on the expression and activation of phosphatases such as CDC25. PLK1 activates CDC25 which in turn activates the CDK1 and forms an active complex with cyclin B. Cyclin B-CDK1 induce expression of certain factors necessary in the later stages of mitosis such as survivin and condensin [30]. Here we show that overexpression of SLAMF3 specifically inhibits the expression of CDC25 as shown by Q-PCR and western blotting (Supplementary Figure 5A, 5B). This observation confirms the suppressor effect of SLAMF3 on pathways that control cell cycle progression. The observations presented here allow us to identify at least one mechanism by which SLAMF3 controls cell cycle progression of cancerous cells by inhibiting PLK1 expression and phosphorylation. Second, by inhibiting CDC25 expression and activation, SLAMF3 controls CDK1-cyclin B activation. More importantly, in nine patients from our HCC cohort, we confirmed the high PLK1 expression in T samples compared to its low expression in pT samples. This result is similar to that of He et al., (2009) [15] and suggest that the rate of PLK1 expression could be a molecular marker of the HCC progression and the aggressiveness. A strict inverse correlation, (9/9, 100%) was obtained between expression of SLAMF3 and PLK1. In HCC samples, when PLK1 mRNA level was high, the levels of SLAMF3 was very less or not detectable. Based on METAVIR stage and clinico-biological data of patients, all patients with advanced fibrosis (score > F2) expressed high levels of PLK1 and undetectable levels of SLAMF3. Our results suggest that the expression of SLAMF3 could be considered as a marker of HCC as its expression was inversely correlated to that of PLK1. No significant correlation was detected between the expression of both SLAMF3/PLK1 and HCC etiology. Additional patients need to be analyzed in order to confirm the specificity of SLAMF3 expression in different etiologies such as viral hepatitis, NASH and alcoholic HCC.

Finally, in the present work we also highlight the mechanisms by which the SLAMF3, acts as a tumor repressor, controls the HCC cell proliferation and tumor progression. Overexpression of SLAMF3 inhibited MAPK/ERK1/2 phosphorylation in HCC wild type cells where ERK1/2 was constitutively activated [10]. Among the many roles, one role of the MAPK ERK cascade is the regulation of G2/M and mitosis progression. Indeed, all components of the cascade were shown to undergo activation during the late G2 and M phases of the cell cycle [31–33]. Several molecular mechanisms have been implicated in the regulation of G2/M by the MAPK/ERK cascade, including the phosphorylation of centromere protein E [34], SWI–SNF [35], Myt1 [36] as well as the indirect activation of PLK1 and Cdc2 [37]. Taken together, the SLAMF3-induced reduction in the activity of MAPK/ERK may control mitosis by inhibition of PLK1 expression and activation. This effect may be accentuated by the repressor effect of RB on PLK1 promoter. Indeed, PLK1 is a target of the RB suppressor pathway and several reports proposed that activation of RB, by its hypophosphorylation, mediate attenuation of PLK1 by controlling PLK1 promoter activity [17]. Our observations suggest that the induction of high level expression of SLAMF3 could be one of potent therapeutic strategy to control tumor progression. Thus, additional studies are needed to identify the molecular partners of hepatic SLAMF3 and study its implications in tumor-suppressing functions.

## MATERIALS AND METHODS

### Patient samples and cell culture

Thirteen pairs (*n* = 13) of tumor (T) samples and matched peritumoral (pT) samples were obtained from HCC patients undergoing surgical resection at Amiens University Hospital (Amiens, France). Our protocol was approved by the local independent ethics committee (Comité de Protection des Personnes (CPP) Nord-Ouest, Amiens, France). Patients were provided with information on the study procedures and objectives and gave their written consent to participation. The clinical and biological information of patients are summarized in Table 1. Total mRNAs and proteins were extracted using specific kits and used for further analysis.

**Table 1:** Clinical and biological parameters of HCC patients

Patients	Age	Sex	Tumor size (cm)	Liver cirrhosis (Y/N)	NASH (Y/N)	Virus	METAVIR	Plk1(T/pT)	SLAMF3(T/pT)	CD3 (in T)	CD64 (in T)
#1	71	M	4,5 × 3 × 3	Y	Y	N	score	28	0.04	−	−
#2	84	M	4 × 3 × 3	Y	N	N	A1F3	3	0.4	−	−
#3	65	M	24 × 18 × 15	N	N	N	A1F3	40	0.05	−	−
#4	74	M	7 × 7 × 6	Y	Y	N	A1F1	3	0.5	−	−
#5	63	M	13 × 12 × 10	N	N	N	A1F4	18	0.1	−	−
#6	56	M	1, 8 × 1,5 × 1, 5	Y	Y	N	A1F1	10	0.3	−	−
#7	59	M	5 × 5 × 5	Y	N	N	A1F4	2	0.3	−	−
#8	61	M	5 × 4 × 4	N	Y	N	A1F4	2	0.5	−	−
#9	NC	M	NC	NC	NC	NC	A1F2	3	0.3	−	−
#10	78	M	7 × 7 × 4	Y	Y	N	NC	ND	50	+	+
#11	70	M	4, 5 × 3, 5 × 4, 5	Y	N	N	A1F2-3	ND	36	+	+
#12	NC	M	NC	NC	NC	NC	A1-A2/F3-	ND	128	+	+
#13	78	M	3 × 2, 5	Y	N	N	F4	ND	5	+	+

Age Years; Sex (M: Male); NC non-communicated; Y: Yes N: No; N: No for viral infection; METAVIR Score A: Area, F Fibrosis; T: Tumor, pT peri-tumor samples.

Human HCC-derived cell line Huh-7 was obtained from virology department (Pr G. Duverlie, CURS, EA 4294 (virology laboratory, CHU Sud, Amiens). Hepatocarcinoma cell lines HepG2, Hep3B, SNU 398 and SNU 449 were obtained from ATCC. The cells were maintained in DMEM (Life Technologies/Invitrogen, Saint Aubin, France) supplemented with 10% fetal calf serum (FCS) (PAA, Velizy-Villacoublay, France) and 1% penicillin/streptomycin (Life Technologies/Invitrogen, Saint Aubin, France). Huh-7 cells stably transfected with RB-shRNA were received from Pr A. Galmiche (service de Biochimie, CBH, CHU Sud, Amiens) and were maintained in DMEM supplemented (Life Technologies/Invitrogen, Saint Aubin, France) with puromycin 2 μM (Calbiochem, Merck-Millipore, France). Healthy human primary hepatocytes PHH (Lonza, Basel, Switzerland) were maintained in phenol red and serum-free HBCTM Basal Medium. HBCTM SingleQuots^®^Kit containing 500 μL hEGF, 500 μL transferrin, 500 μL hydrocortisone, 10 mL bovine serum albumin (BSA), 500 μL ascorbic acid, 500 μL GA-1000 and 500 μL insulin was added to basal medium (Lonza, Basel, Switzerland).

### Antibodies and reagents

For western blot (WB) analysis, anti-SLAMF3 (Goat polyclonal anti-SLAMF3, clone K-12, from Santa Cruz Biotechnology (Heidelberg, Germany) were used. Rabbit antibodies directed against human PLK1 (clone 208G4) and phospho-Plk1 (Thr 210) (clone 208G4), anti-CDC25 (5H9), anti-RB (Clone G3-245, BD-Pharmingen, France), anti-ERK 1/2 and phospho ERK 1/2 (Phospho-p44/42 MAPK ERK 1/2, Thr202/Tyr204, clone 9101), HRP-conjugated anti-rabbit antibodies and HRP-conjugated anti-goat antibodies were purchased from Cell Signaling Technology (Beverly, MA, USA). Anti-actin antibody (clone C-11), used as a control for WB was from Santa Cruz Biotechnology (Heidelberg, Germany). For flow cytometry analysis, the Fluorescein isothiocyanate (FITC) and Phycoerythrin (PE) conjugated monoclonal anti-SLAMF3 (clone HLy9.1.25) and the matched isotype IgG1 were purchased from AbDSerotec (Colmar, France).

### mRNA extraction, quantitative PCR, sequencing and plasmid construction

Total mRNA was extracted using RNeasy kit (Qiagen) and RT-PCR was performed using 100 ng of total RNA. Quantitative PCR was performed according to the Taqman Gene Expression protocol (Applied Biosystems) using the following primers for SLAMF3: forward 5′- tgg gac taa gag cct ctg gaa a-3′, reverse 5′-aca gag att gag aac gtc atc tgg-3′ and MGB probe with 6-FAM (5′-ccc caa cag tgg tgt c-3′). The transcription of GAPDH was measured as an endogenous housekeeping control. The hepatic SLAMF3 cloned in Mammalian expression vector pBud CE 4.1 vector (Invitrogen) previously described [10]. For SLAMF3 overexpression, cells (0.3 × 10^6^) were first seeded into six-well plates 24 h prior to transfection and transfected with 0.8 μg of plasmid DNA using the FuGENE HD Transfection Reagent Kit (Roche, Meylan, France) according to the manufacturer's instructions. Cells were incubated for 48 h at 37°C before analysis of SLAMF3 expression by mRNA quantification, flow cytometry and WB. For Q-PCR quantification, the following primers were used: PLK1 For-aga aga ccc tgt gtg gga ct, Rev-tca aaa ggt ggt ttg ccc ac; CDC25 For-att ctc atc tga gcg tgg gc, Rev-act cct ttg tag ccg cct ttc, and GAPDH For-aag gtg aag gtc gga gtc aa, Rev-ctt gac ggt gcc atg gaa tt.

### Western blot analysis

Cells (10^6^ per assay) from Huh-7 cultures and primary tissues were lysed in Nonidet P40 (NP40) buffer (1% NP40, 50 mMTris pH 7.5, 10% glycerol, 150 mMNaCL, 1 mM EDTA, 100 mM Na3VO6, 0.5 mM phenylmethanesulphonyl fluoride (PMSF), 5 mg/ml aprotinin, 5 mg/ml leupeptin and 2 mg/ml pepstatin) containing protease and phosphatase inhibitors (Roche). Equal amounts of each protein sample were separated by electrophoresis on SDS-PAGE, blotted onto nitrocellulose membranes (Bio-Rad, Munich, Germany) and blotted with antibodies against SLAMF3, PLK1, RB, CDC25, ERK and actin. Blots were developed with the enhanced chemiluminescence (ECL) system (Amersham Pharmacia Biotech).

### Cell sorting and flow cytometry analysis

Transfected cells (24 h post-transfection) were collected in cold PBS/0.01% sodium azide/0.5% BSA, washed and incubated with fluorescent-conjugated antibody anti-SLAMF3 (clone HLy9.1.25, AbDSerotec, Colmar, France) for 30 min at 4°C. Cells were sorted for SLAMF3 using a FACSAria flow cytomer running FACSDiva software (BD Biosciences, Le Pont de Claix, France). Cell purity of both sub-populations was checked by flow cytometry and was upto 98%. SLAMF3^−/Low^ expressed less than 1% while SLAMF3^+/High^ expressed more than 90% of SLAMF3.

For staining, cells were collected in cold PBS/0.01% sodium azide/0.5% BSA, washed and incubated with fluorescent-conjugated primary or isotype-matched antibodies for 20 min at 4°C. Following extensive washing (in PBS/0.01% sodium azide), cells were fixed (in 1% paraformaldehyde) and 5000 viable events were analyzed in the cytometer.

### Hepatocyte proliferation, cell cycle analysis and giemsa staining

The bromure 3-(4,5-dimethylthiazol- 2-yl)-2,5-diphenyl tetrazolium test (MTT) was used to check the anti-proliferative effect of SLAMF3 expression in HCC cells. Cells (sorted Huh-7 SLAMF3^−/Low^ and SLAMF3^+/High^) were seeded at 10^4^ cells/well in 96-well plates. At 24, 48 and 72 h, cells were rinsed and exposed for 1 h to a solution of thiozalyl blue tetrazolium bromide suspended at a concentration of 0.5 mg/ml in colorless culture medium (MTT assay kit from Sigma-Aldrich, St Quentin Fallavier, France). Reduced purple formazan crystals were extracted with DMSO and analyzed at a wavelength of 560 nm.

For cell cycle analysis, cells were seeded at the density of 1 × 10^6^ cells, and cell cycle distribution was analyzed by flow cytometry at 48 h after the transfection of SLAMF3 plasmid. After washing twice with PBS, cells were harvested and collected by centrifugation, and treated with ribonuclease RNase (Interchim) followed by fixation in ice-cold 70% ethanol at −20°C overnight. Then, cells were collected and stained with 100 μl Propidium iodide PI (50 μg/ml) and RNase (10 μg/ml) solution for 30 min in the dark followed by cell cycle analysis. To estimate nuclear and cytoplasm sizes of cells, 48 h cultured cells from Huh-7 SLAMF3^−/Low^ and SLAMF3^+/High^ subpopulations were fixed after 48 h in methanol for 10 min and air-dried. Then they were immersed with the Giemsa solution for 45 min, washed with distilled water and air-dried.

### Statistical analysis

Independent Student's *t*-test was used to compare mRNA expression in T and pT samples. Unless otherwise stated, results are expressed as the mean ± SD. Statistical analyses were performed with Prism software (version 4.0, GraphPad Inc., San Diego, CA, USA). The threshold for statistical significant was set to *p* < 0.05 for all analyses.

## SUPPLEMENTARY MATERIALS FIGURES



## References

[1] Llovet JM, Burroughs A, Bruix J (2003). Hepatocellular carcinoma. Lancet.

[2] Rehermann B, Nascimbeni M (2005). Immunology of hepatitis B virus and hepatitis C virus infection. Nat Rev Immunol.

[3] Lu ZL, Luo DZ, Wen JM (2005). Expression and significance of tumor-related genes in HCC. World J Gastroenterol.

[4] Shaw RJ, Cantley LC (2006). Ras, PI(3)K and mTOR signalling controls tumour cell growth. Nature.

[5] He X, Zhu Z, Johnson C, Stoops J, Eaker AE, Bowen W, DeFrances MC (2008). PIK3IP1, a negative regulator of PI3K, suppresses the development of hepatocellular carcinoma. Cancer Res.

[6] Calvisi DF, Ladu S, Gorden A, Farina M, Conner EA, Lee JS, Factor VM, Thorgeirsson SS (2006). Ubiquitous activation of Ras and Jak/Stat pathways in human HCC. Gastroenterology.

[7] Hwang YH, Choi JY, Kim S, Chung ES, Kim T, Koh SS, Lee B, Bae SH, Kim J, Park YM (2004). Over-expression of c-raf-1 proto-oncogene in liver cirrhosis and hepatocellular carcinoma. Hepatol Res.

[8] Feng DY, Zheng H, Tan Y, Cheng RX (2001). Effect of phosphorylation of MAPK and Stat3 and expression of c-fos and c-jun proteins on hepatocarcinogenesis and their clinical significance. World J Gastroenterol.

[9] Veillette A, Dong Z, Perez-Quintero LA, Zhong MC, Cruz-Munoz ME (2009). Importance and mechanism of ‘switch’ function of SAP family adapters. Immunol Rev.

[10] Marcq I, Nyga R, Cartier F, Amrathlal RS, Ossart C, Ouled-Haddou H, Ghamlouch H, Galmiche A, Chatelain D, Lamotte L, Debuysscher V, Fuentes V, Nguyen-Khac E (2013). Identification of SLAMF3 (CD229) as an inhibitor of hepatocellular carcinoma cell proliferation and tumour progression. PLoS One.

[11] Uchiumi T, Longo DL, Ferris DK (1997). Cell cycle regulation of the human polo-like kinase (PLK) promoter. J Biol Chem.

[12] Barr FA, Sillje HH, Nigg EA (2004). Polo-like kinases and the orchestration of cell division. Nat Rev Mol Cell Biol.

[13] Xie S, Xie B, Lee MY, Dai W (2005). Regulation of cell cycle checkpoints by polo-like kinases. Oncogene.

[14] Sunkel CE, Glover DM (1988). polo, a mitotic mutant of Drosophila displaying abnormal spindle poles. J Cell Sci.

[15] He ZL, Zheng H, Lin H, Miao XY, Zhong DW (2009). Overexpression of polo-like kinase1 predicts a poor prognosis in hepatocellular carcinoma patients. World J Gastroenterol.

[16] He Z, Wu J, Dang H, Lin H, Zheng H, Zhong D (2011). Polo-like kinase 1 contributes to the tumorigenicity of BEL-7402 hepatoma cells via regulation of Survivin expression. Cancer Lett.

[17] Gunawardena RW, Siddiqui H, Solomon DA, Mayhew CN, Held J, Angus SP, Knudsen ES (2004). Hierarchical requirement of SWI/SNF in retinoblastoma tumor suppressor-mediated repression of Plk1. J Biol Chem.

[18] Burkhart DL, Sage J (2008). Cellular mechanisms of tumour suppression by the retinoblastoma gene. Nat Rev Cancer.

[19] Singh S, Johnson J, Chellappan S (2010). Small molecule regulators of Rb-E2F pathway as modulators of transcription. Biochim Biophys Acta.

[20] Viatour P, Ehmer U, Saddic LA, Dorrell C, Andersen JB, Lin C, Zmoos AF, Mazur PK, Schaffer BE, Ostermeier A, Vogel H, Sylvester KG, Thorgeirsson SS (2011). Notch signaling inhibits hepatocellular carcinoma following inactivation of the RB pathway. J Exp Med.

[21] Lee TK, Man K, Ling MT, Wang XH, Wong YC, Lo CM, Poon RT, Ng IO, Fan ST (2003). Over-expression of Id-1 induces cell proliferation in hepatocellular carcinoma through inactivation of p16INK4a/RB pathway. Carcinogenesis.

[22] Engel P, Eck MJ, Terhorst C (2003). The SAP and SLAM families in immune responses and X-linked lymphoproliferative disease. Nat Rev Immunol.

[23] Liu X, Erikson RL (2002). Activation of Cdc2/cyclin B and inhibition of centrosome amplification in cells depleted of Plk1 by siRNA. Proc Natl Acad Sci USA.

[24] Liu X, Erikson RL (2003). Polo-like kinase (Plk)1 depletion induces apoptosis in cancer cells. Proc Natl Acad Sci USA.

[25] Spankuch-Schmitt B, Bereiter-Hahn J, Kaufmann M, Strebhardt K (2002). Effect of RNA silencing of polo-like kinase-1 (PLK1) on apoptosis and spindle formation in human cancer cells. J Natl Cancer Inst.

[26] Yuan J, Kramer A, Eckerdt F, Kaufmann M, Strebhardt K (2002). Efficient internalization of the polo-box of polo-like kinase 1 fused to an Antennapedia peptide results in inhibition of cancer cell proliferation. Cancer Res.

[27] Strebhardt K (2010). Multifaceted polo-like kinases: drug targets and antitargets for cancer therapy. Nat Rev Drug Discov.

[28] Degenhardt Y, Lampkin T (2010). Targeting Polo-like kinase in cancer therapy. Clin Cancer Res.

[29] Brandwein JM (2015). Targeting polo-like kinase 1 in acute myeloid leukemia. Ther Adv Hematol.

[30] Castedo M, Perfettini JL, Roumier T, Andreau K, Medema R, Kroemer G (2004). Cell death by mitotic catastrophe: a molecular definition. Oncogene.

[31] Tamemoto H, Kadowaki T, Tobe K, Ueki K, Izumi T, Chatani Y, Kohno M, Kasuga M, Yazaki Y, Akanuma Y (1992). Biphasic activation of two mitogen-activated protein kinases during the cell cycle in mammalian cells. J Biol Chem.

[32] Edelmann HM, Kuhne C, Petritsch C, Ballou LM (1996). Cell cycle regulation of p70 S6 kinase and p42/p44 mitogen-activated protein kinases in Swiss mouse 3T3 fibroblasts. J Biol Chem.

[33] Shapiro PS, Vaisberg E, Hunt AJ, Tolwinski NS, Whalen AM, McIntosh JR, Ahn NG (1998). Activation of the MKK/ERK pathway during somatic cell mitosis: direct interactions of active ERK with kinetochores and regulation of the mitotic 3F3/2 phosphoantigen. J Cell Biol.

[34] Zecevic M, Catling AD, Eblen ST, Renzi L, Hittle JC, Yen TJ, Gorbsky GJ, Weber MJ (1998). Active MAP kinase in mitosis: localization at kinetochores and association with the motor protein CENP-E. J Cell Biol.

[35] Sif S, Stukenberg PT, Kirschner MW, Kingston RE (1998). Mitotic inactivation of a human SWI/SNF chromatin remodeling complex. Genes Dev.

[36] Palmer A, Gavin AC, Nebreda AR (1998). A link between MAP kinase and p34(cdc2)/cyclin B during oocyte maturation: p90(rsk) phosphorylates and inactivates the p34(cdc2) inhibitory kinase Myt1. EMBO J.

[37] Liu X, Yan S, Zhou T, Terada Y, Erikson RL (2004). The MAP kinase pathway is required for entry into mitosis and cell survival. Oncogene.

