# Supplementary material for: RB/PLK1-dependent induced pathway by SLAMF3 expression inhibits mitosis and control hepatocarcinoma cell proliferation
Source: Oncotarget. 2016 Jan 20;7(9):9832–43. doi: 10.18632/oncotarget.6954 (PMC4891087; doi:10.18632/oncotarget.6954)
Supplement: Supplementary file 1 [file oncotarget-07-09832-s001.pdf]

RB/PLK1-dependent induced pathway by SLAMF3 expression inhibits mitosis and control hepatocarcinoma cell proliferation

Supplementary Materials

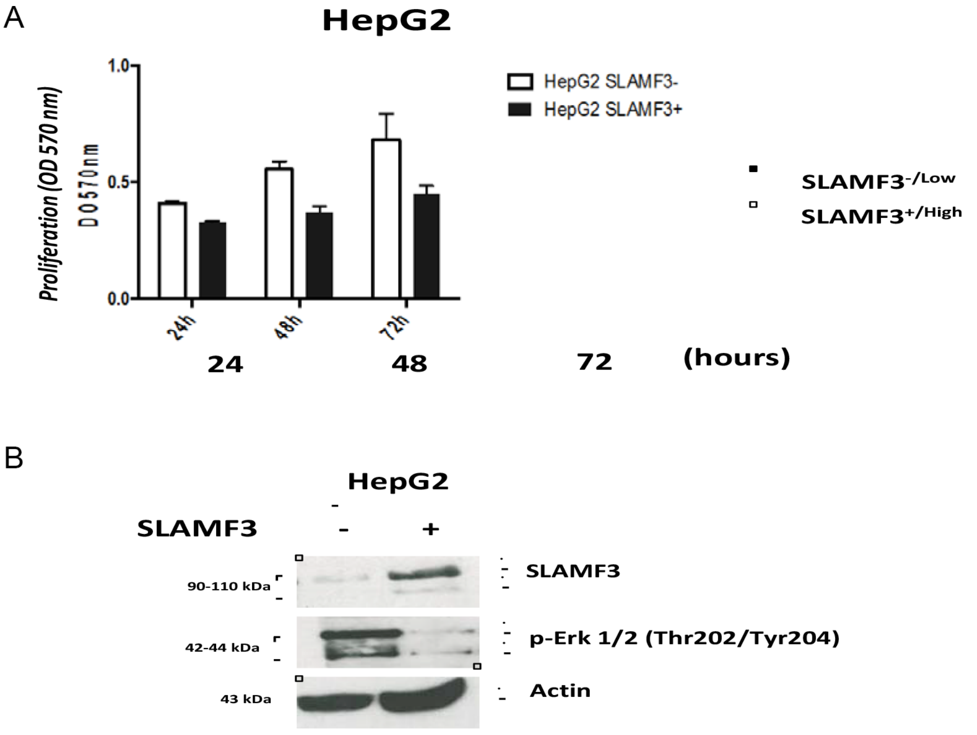

Supplementary Figure S1: High level expression of SLAMF3 inhibits HepG2 proliferation and MAPK ERK1/2 activation. (**A**) Proliferation was determined by MTT test after 24, 48 and 72 h cultures of sorted SLAMF3$^{+/high}$ and SLAMF3$^{-/low}$ sub-populations after introduction of SLAMF3 coding plasmid in cells from HepG2 cell line. The results are presented as the mean SD from 3 independent experiments ($n = 3$; ***$p < 0.005$). (**B**) SLAMF3 expression level and phospho-ERK1/2 proteins were detected by Western blot analysis in SLAMF3$^{+/high}$ and SLAMF3$^{-/low}$ sub-populations of HepG2 cells. Detection of actin was used as control. One representative result from 3 independent experiments was shown.

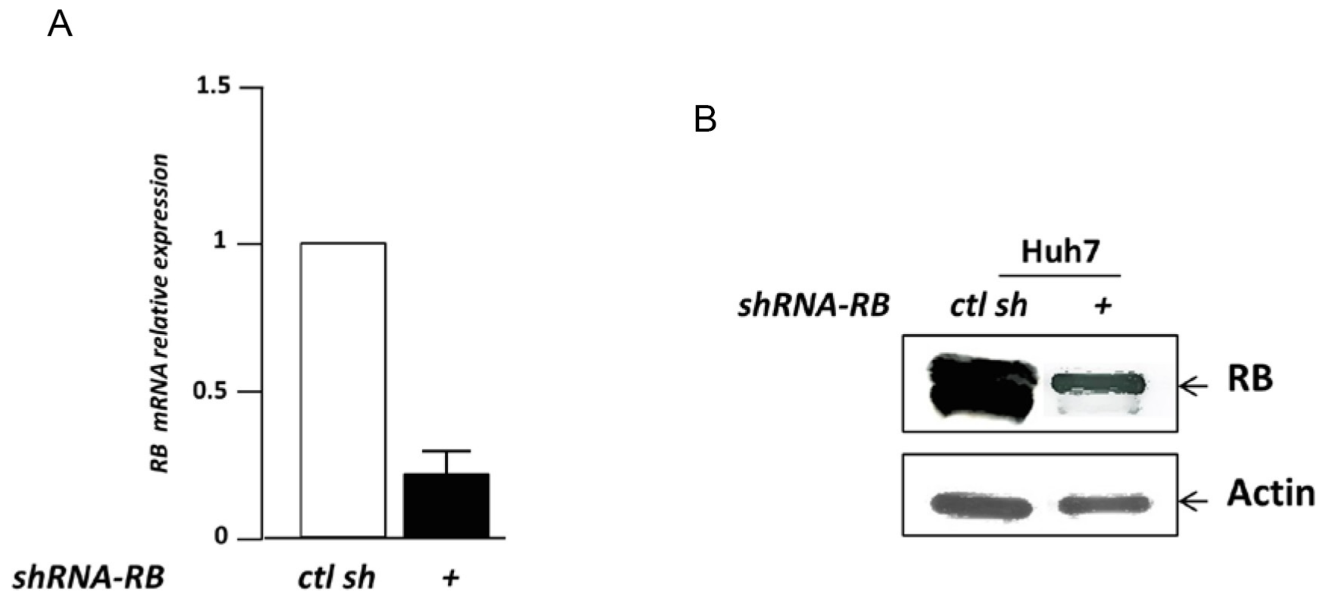

Supplementary Figure S2: Specific shRNA directed against RB inhibited the expression of RB. (**A**) RB mRNA and (**B**) RB protein. The stable shRNA transfection achieved inhibition of RB as transcripts and protein. The RB expression was checked in Huh-7 stably transfected by shRNA RB and control shRNA (*ctl sh*); Detection of actin was used as control. One representative result from 3 independent experiments is shown.

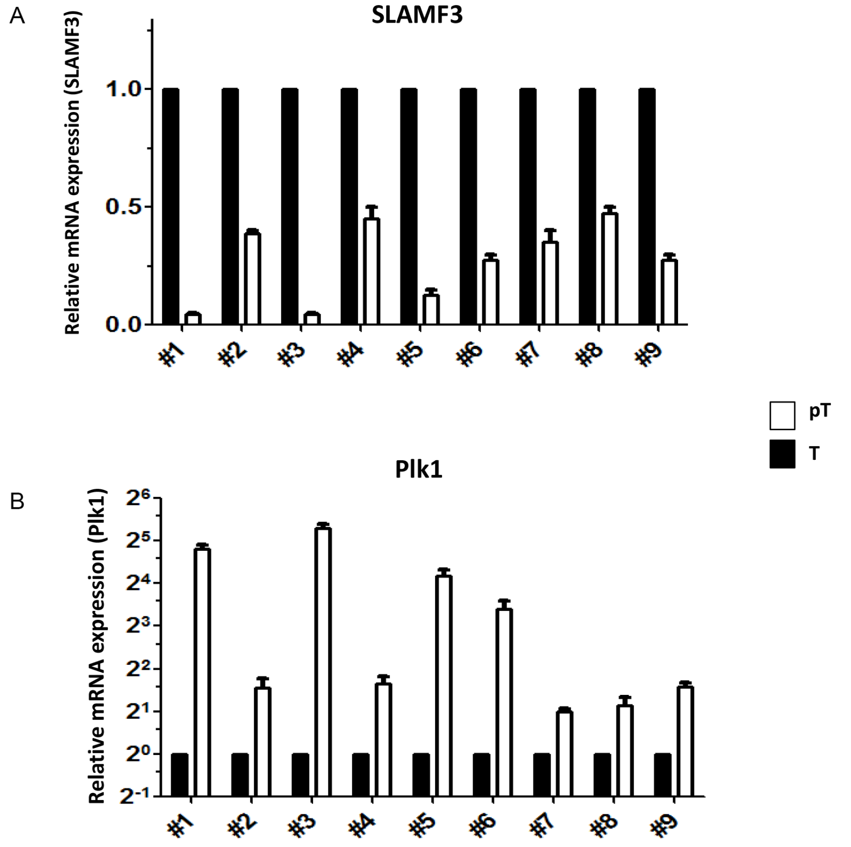

Supplementary Figure S3: Quantification of SLAMF3 and PLK1 transcripts in patients with HCC. The mRNA quantification by Q-PCR of (**A**) SLAMF3 and (**B**) PLK1 was carried out in nine ($n = 9$) of paired pT /T samples from HCC patients. Results are presented for each patient independently as mean of three independent quantifications of each sample and presented as mean +/− SD.

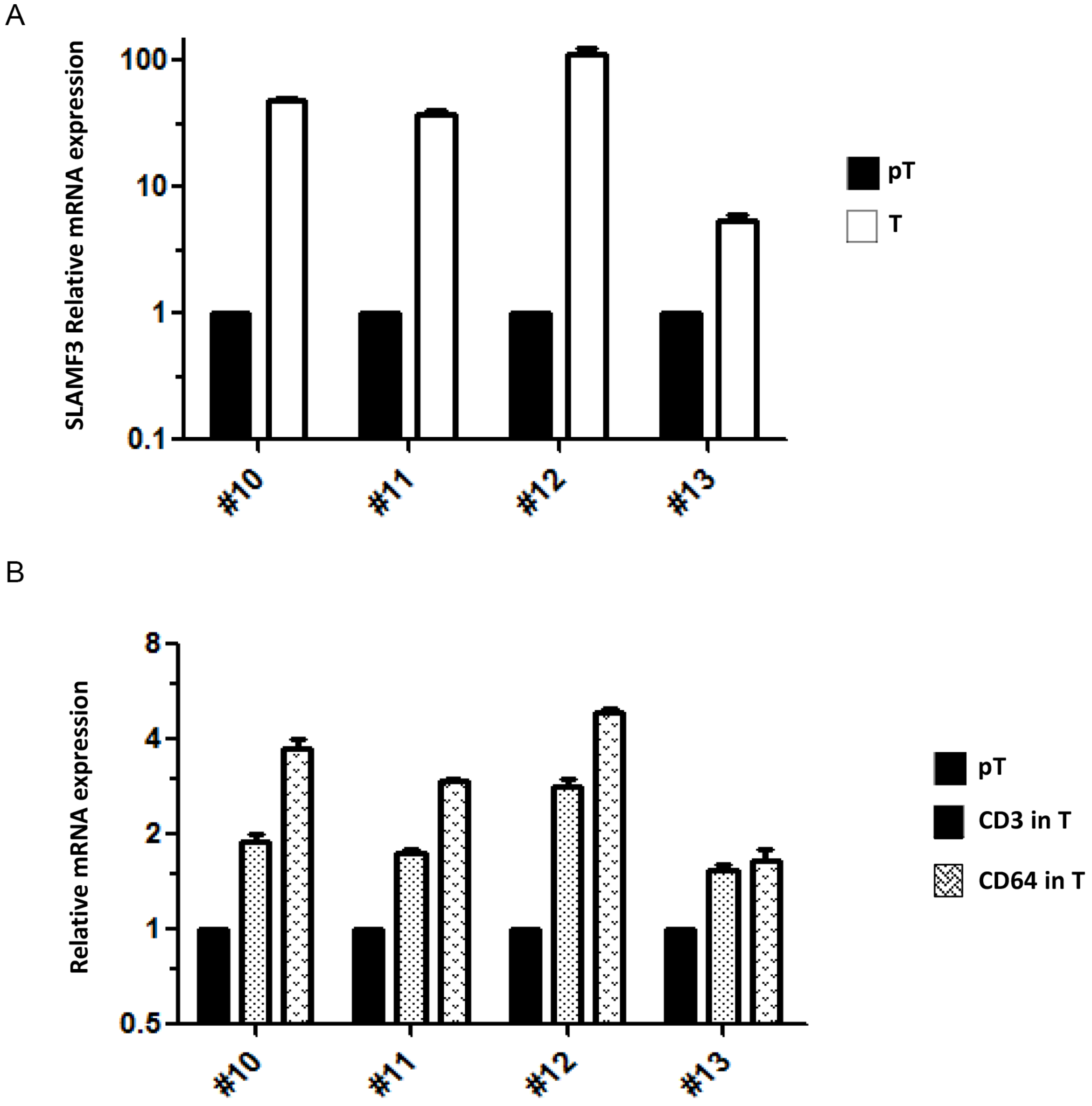

Supplementary Figure S4: Tumor samples with high SLAMF3 expression levels were checked for lymphocytes and macrophages infiltration. (**A**) Relative mRNA level of SLAMF3 in tumor samples (T) from four samples in which CD3 and CD64 transcripts, markers for T lymphocytes and macrophages, respectively, were quantified as control (**B**). Results were presented for each patient independently as mean of three independent quantifications of each sample and presented as mean +/− SD.

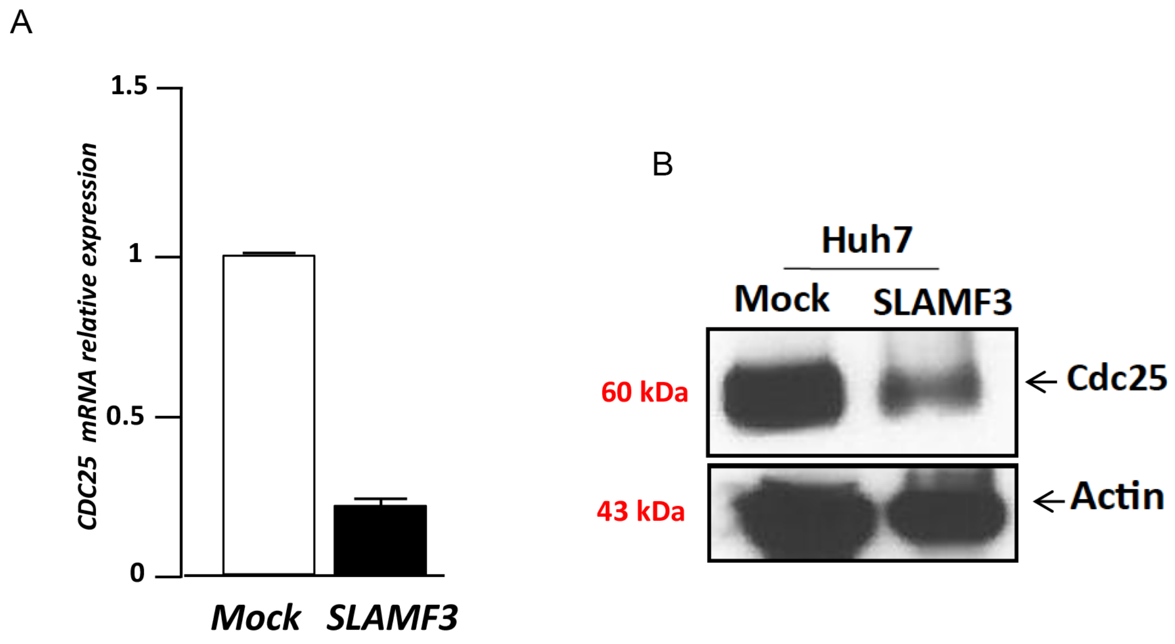

Supplementary Figure S5: High level expression of SLAMF3 decreased Cdc25 expression. The Cdc25 expression was checked in Huh-7 overexpressing SLAMF3 (SLAMF3) and in mock (Huh-7 transfected by free plasmid). Actin was used as protein control. One representative result from 2 independent experiments was shown.
